# The Impact of Sanming Healthcare Reform on Antibiotic Appropriate Use in County Hospitals in China

**DOI:** 10.3389/fpubh.2022.936719

**Published:** 2022-06-27

**Authors:** Lin Hu, Mengyuan Fu, Haishaerjiang Wushouer, Bingyu Ni, Huangqianyu Li, Xiaodong Guan, Luwen Shi

**Affiliations:** ^1^Department of Pharmacy Administration and Clinical Pharmacy, School of Pharmaceutical Sciences, Peking University, Beijing, China; ^2^International Research Center for Medicinal Administration, Peking University, Beijing, China

**Keywords:** systemic reform, governance structure, antibiotic use, public hospitals, healthcare reform

## Abstract

**Background:**

The excessive use of resources and poor quality of care are great concerns worldwide, particularly in China. In 2013, a model of systematic reforms was developed in Sanming to address the inefficiency and waste in public hospitals. However, limited empirical studies have evaluated the effect of Sanming healthcare reform on antibiotic appropriate use. This study aims to evaluate the impact of the healthcare reform on the appropriate use of antibiotics in county-level public hospitals in Sanming, China.

**Methods:**

We conducted a retrospective observational study exploring trends in antibiotic use with an interrupted time series design. We selected three county-level hospitals in Sanming and extracted outpatient prescriptions of the Departments of Internal Medicine and the Department of Pediatrics between January 2011 and December 2017. Acute Upper Respiratory Tract Infection (AURI), Acute Bronchitis (AB) and Community Acquired Pneumonia (CAP) were selected as the sample diseases for our analysis. The primary outcome was the percentage of prescriptions conformed with standard treatment guidelines (STGs).

**Results:**

A total of 142,180 prescriptions were included in the analysis. During the study period, the percentage of antibiotics prescriptions conformed with STGs boosted from 32.4% in 2011 to 82.3% in 2017. Moreover, after the reform, the rate of prescriptions that conformed with STGs showed significant increasing trends in children with AURI (β = 1.624, *p* < 0.001), children with AB (β = 3.123, *p* < 0.001), adult with AB (β = 1.665, *p* < 0.001), children with CAP (β = 3.123, *p* < 0.001), adult with CAP (β = 4.385, *p* < 0.001), but not in adult patients with AURI (β = −0.360, *p* = 0.022).

**Conclusion:**

Our study confirmed that the Sanming healthcare reform helped to promote the appropriate use of antibiotics in county-level hospitals. This systematic approach to healthcare reform, characterized by an effective governance structure, dynamic financial compensation mechanisms, and specialized drug stewardship, is promising for future public hospital reforms.

## Background

The excessive use of resources and poor quality of care are great concerns for health systems worldwide (1–3). In recent years, various policies have been implemented to improve healthcare performance (4, 5) and manage healthcare expenditures (6, 7). Among these measures, public hospital reform showed promosing results, especially in many low- and middle-income countries (8, 9). In 2012, to reduce over-prescription and to control the rapid growth of healthcare expenditure, the Chinese government launched a nationwide reform for county-level public hospitals (10). The main measures included improving the reimbursement mechanism, and restructuring the management system of public hospitals (9).

Among many pilot public hospital reforms in China, the model developed by Sanming City showed great potentials and became one of the leading hospital reform models (11, 12). Sanming city rolled out its comprehensive healthcare system reform in all public hospitals in January 2013. The reform covered three crucial aspects (13). First, Sanming systematically reformed its governance structure, centralizing and granting the authority to set health policies and oversee public hospitals into one commission (11, 12). The commission issued guidelines for infrastructure construction, equipment and drug procurement, hospital management, and physician compensation. Second, Sanming standardized the drug procurement process to reduce drug cost by implementing Zero Mark-up Drug Policy (ZMDP) and Centralized Procurement of Medicine Policy (CPMP) (12). ZMDP was introduced in February 2013 to alleviate hospitals' heavy reliance on drug sales by removing mark-ups for any drug sold by public hospitals. In June 2013, CPMP was implemented to strengthen the bargaining power on the demand's side and streamline supply chains of medicines. Third, Sanming altered physician compensation mechanisms by breaking the link between physician income and “profits” resulting from their services. Instead, physicians were paid a higher basic salary and a performance-based bonus (11).

Studies evaluating the impacts of the Sanming healthcare reform found that the Sanming model significantly reduced health expenditures, particularly drug expenses, without notably disadvantaging the quality and efficiency of care (11–14). However, no empirical study has evaluated the effect of Sanming healthcare reform on the appropriate use of drugs, especially antibiotics. In this study, we aimed to explore the impact of the Sanming healthcare reform on the appropriate use of antibiotics in county-level public hospitals within the city's administration.

## Materials and Methods

### Study Design and Settings

We employed a quasi-natural experiment design with an interrupted time series (ITS) approach in this study. Sanming is a prefecture-level city in Fujian province, with a residential population of 2.74 million across a geographic area of 22,965 square kilometers in 2012 (15). The Gross Domestic Product (GDP) per capita of Sanming in 2012 was about $8,500, which ranked the fifth among the nine cities of Fujian province (11). And the total health expenditure in Sanming accounted for 4.45% of its gross domestic product (GDP). Approximately 1.65 registered physicians, 2.02 nurses, and 4.31 hospital beds per 1000 people (15) were available in Sanming in 2012.

### Study Participants

#### Sampling

We randomly selected three out of 12 county-level hospitals in Sanming city. Withinin each hospital, only the departments of internal medicine and pediatrics were included in the analysis because of their high frequency of antibiotics use (16).

All the outpatient prescriptions prescribed between January 2011 and December 2017 were identified and retrieved from the electronic medical records. For manual prescriptions during times when the electronic database did not go fully online, 50 outpatient prescriptions were randomly selected from patient encounters that took place on the second Tuesday of each month from each sample department. If the quantity of visits was not sufficient, the records from the following day were collected.

Acute Upper Respiratory Tract Infection (AURI), Acute Bronchitis (AB), and Community Acquired Pneumonia (CAP) were included as the sample diseases to assess the trends in antibiotics use. These were diseases for which antibiotics were most commonly prescribed, with 60–80% improper antibiotic prescription in outpatient settings in China (17, 18). Prescriptions showing a diagnosis of only one of the above three diseases were eligible for this study. To assess potential differences in antibiotics prescribing by age, the data were sub-categorized into two age groups: children (0–17 years old) and adults (18–64 years old).

#### Data Collection and Inclusion

Prescription information including department name, visit date, demographics, diagnoses and medications prescribed were collected, and the data were digitally transferred and verified by two investigators. Outpatient visits with at least one drug prescribed were eligible for inclusion. Vaccines and herbal medicines were excluded.

### Measurements

#### Definition of Antibiotics

We classified medicines according to the Anatomical Therapeutic and Chemical (ATC) classification codes, as recommended by the World Health Organization Collaborating Centre for Drug Statistic Methodology (19). Drugs classified as “J01” (antibacterials for systemic use) were defined as antibiotics in this study.

#### Outcomes

The appropriateness of antibiotics prescription was assessed based on the national standard treatment guidelines (STGs) (20–24). The primary outcome was the percentage of prescriptions conformed with STGs. The numerator was the number of prescriptions in which all antibiotics prescribed conformed with guidelines, and the denominator was the total number of prescriptions that contained at least one antibiotic. Secondary outcomes included the percentage of presecriptions with at least one antibiotic, and the percentage of presecriptions with two or more antibiotics.

### Statistical Analysis

We conducted descriptive analyses and used an ITS design to assess the impact of the Sanming healthcare reform on the appropriate use of antibiotics in public hospitals. Percentages of prescriptions conformed with STGs before and after the intervention were compared. The first two quarters of 2013 (January 2013 to June 2013), during which most reform measures were introduced in Sanming City, were regarded as the intervention time point for ITS analysis. This study period covered 28 consecutive quarters, consisting of nine quarters before and 19 quarters after the intervention. The following model was applied for the quarterly analysis:


$$\begin{array}{l} Y_{t}=\beta_{0}+\beta_{1}\cdot time_{t}+\beta_{2}\cdot intervention_{t}\\ \;+\beta_{3}\cdot time\_after\_intervention_{t}+\varepsilon_{t} \end{array}$$

The basic model included terms to estimate the baseline level for each outcome (intercept) (β_0_), baseline trend (slope) (β_1_), changes in the level immediately after policy implementation (β_2_), and changes in the trend after the implementation (β_3_). time_t_ is an integer variable indicating the time measured in quarters at time t from the beginning of the study period; intervention_t_ is a dummy indicator representing the presence of the Sanming healthcare reform with a value of 0 before the reform and 1 thereafter; time_after_intervention_t_ is the time after the reform begins to have an impact; and ε_t_ is an estimate of the random error at time *t*. The Durbin-Watson test was performed to estimate the level of residual autocorrelations and the Prais-Winsten auto-regression procedure was used to correct first order serially correlated errors when needed. The seasonal effect will be modified by regarding the current point value as the average value of the current point and previous three data points. The level of statistical significance was defined as *p* < 0.05 (two-sided). We performed all statistical analysis using STATA 15.1.

## Results

### Sample Characteristics

A total of 142,180 prescriptions for sample diseases in three county-level hospitals in Sanming City were eligible for our study, including 124,244 prescriptions for children and 17,936 prescriptions for adults. Among these, 47,834 prescriptions were diagnosed with AURI; 90,064 were diagnosed with AB and 4,282 were diagnosed with CAP (Table 1).

**Table 1:** Sample size and characteristics of prescriptions in county-level hospitals in Sanming.

**Sample size**	**2011**	**2012**	**2013**	**2014**	**2015**	**2016**	**2017**	**Total**
Sample prescriptions	16,601	18,946	20,168	22,301	17,538	24,145	22,481	142,180
**Age group**
Children	14,938	16,627	18,100	19,654	15,226	20,916	18,783	124,244
Adult	1,663	2,319	2,068	2,647	2,312	3,229	3,698	17,936
**Disease group**
Acute upper respiratory tract infection	5,436	6,510	6,987	7,503	6,376	8,199	6,823	47,834
Acute bronchitis	10,858	12,017	12,742	14,235	10,625	15,074	14,513	90,064
Community acquired pneumonia	307	419	439	563	537	872	1,145	4,282

### Descriptive Analysis of Changes in Indicators Measuring Appropriate Antibiotic Use

During the 7-year study period, the percentage of antibiotics prescriptions conformed with STGs experienced a sharp increase from 32.4 to 82.3%. The overall antibiotic prescription rate decreased from 83.5 to 79.3%, and the overall combined antibiotic prescription rate dropped from 14.2 to 6.5% (Table 2).

**Table 2:** Indicators measuring appropriate antibiotic use from 2011 to 2017, by age and disease groups.

**Indicators**	**2011**	**2012**	**2013**	**2014**	**2015**	**2016**	**2017**
**Overall**
STGs compliance rate (%)	32.4	32.9	37.9	56.2	60.8	75.0	82.3
Antibiotic prescription rate (%)	83.5	83.5	83.3	82.6	80.2	80.3	79.3
Combined antibiotic prescription rates (%)	14.2	10.8	11.1	9.0	9.3	8.1	6.5
**Age group**
**Children**
STGs compliance rate (%)	30.7	29.9	35.8	56.2	61.3	76.0	83.0
Antibiotic prescription rate (%)	82.6	82.3	82.2	81.5	78.6	79.5	78.1
Combined antibiotic prescription rates (%)	11.9	7.9	8.9	7.0	4.8	3.7	1.9
**Adult**
STGs compliance rate (%)	45.7	52.4	54.3	56.5	57.6	68.8	78.7
Antibiotic prescription rate (%)	91.1	92.4	93.2	90.8	90.8	85.6	85.2
Combined antibiotic prescription rates (%)	35.1	31.4	29.7	24.6	38.8	37.0	29.8
**Disease group**
**Acute upper respiratory tract infection**
STGs compliance rate (%)	32.7	26.9	29.0	37.4	41.3	45.4	46.3
Antibiotic prescription rate (%)	76.4	75.1	71.8	76.4	73.7	71.2	65.2
Combined antibiotic prescription rates (%)	14.7	12.5	10.0	9.1	11.5	9.5	5.0
**Acute bronchitis**
STGs compliance rate (%)	32.3	35.9	42.4	66.1	72.4	88.8	95.3
Antibiotic prescription rate (%)	87.0	87.9	89.5	85.6	83.5	84.9	85.1
Combined antibiotic prescription rates (%)	13.3	8.6	10.0	7.2	5.5	5.0	4.3
**Community acquired pneumonia**
STGs compliance rate (%)	29.8	26.0	21.8	29.7	37.0	69.3	83.6
Antibiotic prescription rate (%)	86.3	88.1	89.7	86.9	92.6	87.3	89.4
Combined antibiotic prescription rates (%)	37.5	48.9	57.4	53.8	57.4	49.5	42.2

In the subgroup analyses, we detected a significant age variation in antibiotic prescription rates, with consistently higher rates among adults as compared to those in children. Moreover, children also experienced a larger decrease in combined antibiotic rescription rate (10.0%) compared to adults (5.3%). When sectioned by disease groups, CAP was the only disease that showed increases in antibiotic prescription rate (from 86.3 to 89.4%) and the combined antibiotic prescription rate (from 37.5 to 42.2%). Appendix 1 presented descriptive statistics of outcome variables sectioned by age and disease groups.

### ITS Analysis of Changes in STGs Compliance Rate

**Figure 1:**
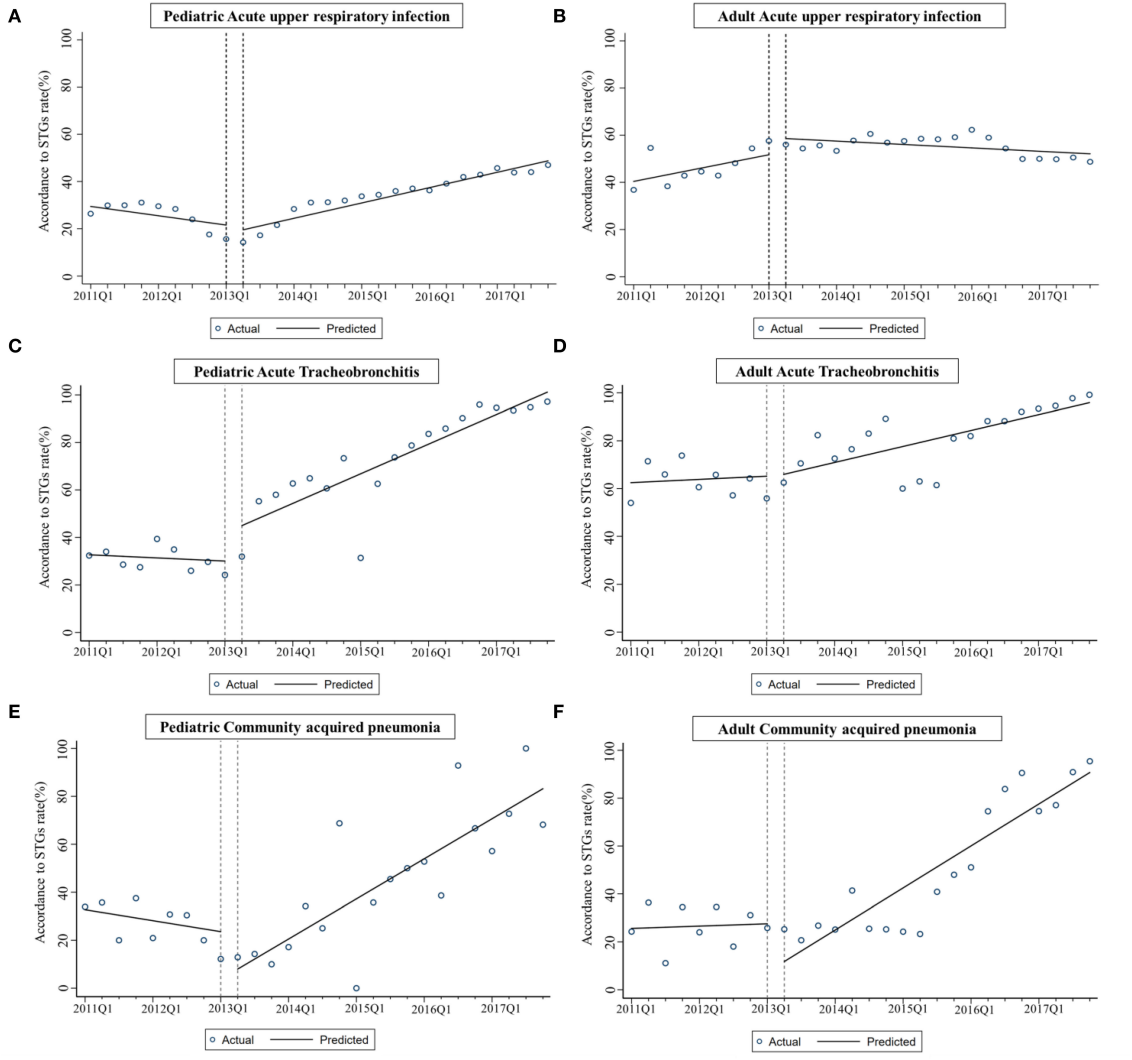
**(A–F)** Estimates from interrupted time-series models of changes in STGs compliance rate for children and adult with three sample diseases after the intervention.

Figure 1 presented the quaterly rate of prescriptions that conformed with STGs from January 2011 to December 2017, sectioned by age and disease groups. The estimated changes in the STGs compliance rate following the policy implementation were presented in Table 3. After the reform, the STGs compliance rate in children with AURI increased rapidly (trend change β = 2.612, *p* = 0.016) and sustained a significant positive trend (β = 1.624, *p* < 0.001). For adult with AURI, there were no obvious level or trend change found in the STGs compliance rate, but a decreasing trend post-intervention was observed (β = −0.360, *p* = 0.022).

**Table 3:** Estimates from interrupted time-series models of changes in STGs compliance rate for children and adult with three sample diseases.

**Rate of accordance to STGs**	**Value (SE)**	***t***	***p***	**95% CI**	
Acute upper respiratory infection*					
**Children**					
Baseline level	29.430 (3.488)	8.44	**<0.001**	22.21	36.65
Baseline trend	−0.988 (0.885)	−1.12	0.276	−2.82	0.84
Level change	−2.555 (2.321)	−1.10	0.282	−7.36	2.25
Trend change	2.612 (1.010)	2.59	**0.016**	0.52	4.70
Post intervention linear trend	1.624 (0.221)	7.35	**<0.001**	1.17	2.08
**Adult**					
Baseline level	40.394 (5.770)	7.00	**<0.001**	28.46	52.33
Baseline trend	1.413 (1.065)	1.33	0.198	−0.79	3.62
Level change	1.560 (1.744)	0.89	0.381	−2.05	5.17
Trend change	−1.773 (1.076)	−1.65	0.113	−4.00	0.45
Post intervention linear trend	−0.360 (0.146)	−2.46	**0.022**	−0.66	−0.06
**Acute bronchitis**					
**Children**					
Baseline level	32.654 (1.655)	19.73	**<0.001**	29.23	36.08
Baseline trend	−0.330 (0.428)	−0.77	0.448	−1.21	0.55
Level change	16.987 (5.990)	2.84	**0.009**	4.60	29.38
Trend change	3.453 (0.565)	6.11	**<0.001**	2.28	4.62
Post intervention linear trend	3.123 (0.370)	8.44	**<0.001**	2.36	3.89
**Adult**					
Baseline level	62.469 (7.541)	8.28	**<0.001**	46.87	78.07
Baseline trend	0.339 (1.451)	0.23	0.818	−2.66	3.34
Level change	7.194 (4.225)	1.70	0.102	−1.55	15.93
Trend change	1.327 (1.494)	0.89	0.384	−1.76	4.42
Post intervention linear trend	1.665 (0.283)	5.89	**<0.001**	1.08	2.25
**Community acquired pneumonia**					
**Children**					
Baseline level	32.739 (2.801)	11.69	**<0.001**	26.95	38.53
Baseline trend	−1.143 (0.606)	−1.89	0.072	−2.40	0.11
Level change	−8.464 (6.611)	−1.28	0.213	−22.14	5.21
Trend change	5.321 (0.736)	7.23	**<0.001**	3.80	6.84
Post intervention linear trend	3.123 (0.393)	10.64	**<0.001**	3.37	4.99
**Adult**					
Baseline level	25.625 (7.485)	3.42	**0.002**	10.14	41.11
Baseline trend	0.241 (1.595)	0.15	0.881	−3.06	3.54
Level change	−12.890 (9.403)	−1.37	0.184	−32.34	6.56
Trend change	4.144 (1.690)	2.45	**0.022**	0.65	7.64
Post intervention linear trend	4.385 (0.540)	8.12	**<0.001**	3.27	5.50

^*^Adjusted for seasonal trend. The bold values indicate the statistical significance values of p < 0.05.

Among patients with AB, the STGs compliance rate in children increased rapidly, with noticeable level and trend change (level change β = 16.987, *P* = 0.009; trend change β = 3.453, *P* < 0.001), while adult patients experienced an increasing trend (β = 1.665, *p* < 0.001) with no obvious level or trend change. Among all patients with CAP, there were no significant level changes observed in STGs compliance rates, while positive trend changes were found in both children (β = 5.321, *P* < 0.001) and adults (β = 4.144, *P* = 0.022) after the reform.

## Discussion

Our study proved that the Sanming healthcare reform has substantially improved the STGs compliance rate of prescriptions with antibiotics and reduced the combined antibiotic prescription rate in county-level hospitals.

Several studies have evaluated the impacts of the public hospital reforms in China (25–29) and showed varying findings of the reforms' influence on antibiotic use in county-level public hospitals. For instance, in Hubei province, the total antibiotic use showed an increasing trend, as measured by procurement volumes on antibiotics, after the healthcare reform (27), which is inconsistent with the findings in Jiangsu province (28) where the antibiotics procurement volumes decreased. These contrasting findings may stem from the differences in geographical areas, medical institution levels and policy setting details. Our findings confirmed that the Sanming public hospital reforms had a positive impact on antibiotic utilization, especially on the structure of drug use. Improvements in the appropriate use antibiotics are attributable to three crucial aspects of the reform.

Sanming applied ZMDP and CPMP to reduce the drug prices. The policies made the medicine procurement process more transparent, reducing the financial incentives to overmedicate of hospitals (12, 30). In addition, centralized procurement increased the opportunity for drugs recommended by the treatment guidelines to be procured and used by the hospitals (31, 32), thereby promoting the appropriate selection of antibiotics for treating infections. However, previous studies (25, 33, 34) suggested that without changing the motivation mechanism behind hospitals' profit-seeking measures, removal of additional mark-ups from drug sales alone failed to sustain improvements achived in the appropriate use of antibiotics.

The Sanming reform distinguished from most pilot reforms by comprehensively revising its governance structure. Hospital directors thus had greater autonomy to run and reform their hospitals (11). In detail, Sanming realigned financial subsidies to hospitals and staff, helping medical income delinked from unmonitored profits (medical rebate). Medical staffs were paid a higher basic salary and a performance-based bonus, further decentivising medical staffs to rely on medical debate for financial gains (13). In addition, a performance measurement system was introduced to rate the quality of clinical care, including the appropriateness of antibiotic use. Meanwhile, the local government held training sessions for professional personnels to ensure that medical staffs were competent to provide specified levels of health care, especially in pediatric (35) and pharmcy departments (36).

The Sanming healthcare reform emphasized Antimicrobial Stewardship (AMS). Public hospitals were assigned with clearly defined tasks to restrict antimicrobial usage with a 3-fold classification system (unrestricted antimicrobials, restricted antimicrobials and special antimicrobials) (30). It mandated the establishment of actualized formulary restriction, established hospital antibiotics consumption surveillance systems, and promoted the review of antibiotic prescriptions by clinical pharmacists and the provision of feedback to prescribers. By prioritizing quality of care and service performance, AMS is a major contributor to improvements in quality of care in Sanming. This echos previous studies (37–40) in that the implementation of AMS significantly reduced the medical service expenditure and the overall drug cost per patient in China.

However, improvements in the appropriate use of antibiotics in China await further enhancements. It is noteworthy that inappropriate antibiotics prescribing in adults with AURI did not progress substantially after the reform. In these prescriptions for adult patients with AURI that failed to conform with STGs, second- and third-generation cephalosporins were the most frequently prescribed. A similar prescribing pattern was observed in previous findings in Chinese hospitals for treating respiratory tract infections (41, 42). This preference for upper-level broad-spectrum cephalosporins for the treatment of uncomplicated respiratory tract infections was also found in the U.S. (43) and European countries (44). Previous studies have shown that the overestimation of penicillin allergy in health records may actually impel some clinicians to replace first-line therapies with cephalosporins or other broad-spectrum antibiotics (45, 46). This phenomenon is still largely overlooked in China. The underlying reasons for the over- and inappropriate use of cephalosporins are complex in real-life. Futher studies focused on other complementary measures to promote appropriate antibiotic prescribing patterns are still needed.

## Limitations

This study has several limitations. First, due to limited resources, only three county-level hospitals were included in this study, risking limiting the generalizability of the estimates and thus study results to other public hospitals. Second, due to the limited availability of electronic medical records, selection bias might have been introduced during the selection of sample prescriptions. Third, although our analysis included prescriptions that showed a diagnosis of only one of the three sample diseases, we cannot assure that all the antibiotics were prescribed solely for the sample diseases in our study. This may contribute to the underestimation of the STGs compliance rates. Fourth, the improvements in antibiotics use in public hospitals may be associated with the Special National Antimicrobial Stewardship Campaign (37). However, this national scheme was launched in April 2011, which was 2 years earlier than our reform. Thus, our results retain a considerable power to suggest that increased appropriate use of antibiotics is attributable to the Sanming healthcare reform. Finally, results in this study could be interpreted as a reflection of the overall impact of healthcare reform measures simultaneously implemented in Sanming, such as ZMDP, Physician Compensation Methods. These are important issues to be addressed in future research.

## Conclusions

Our study provided evidence that the Sanming healthcare reform helped to promote the appropriate use antibiotics in county-level hospitals. This systematic approach to healthcare reform, characterized by an effective governance structure, dynamic financial compensation mechanisms, and specialized drug stewardship, is promising for future public hospital reforms.

## Data Availability Statement

The raw data supporting the conclusions of this article will be made available by the authors, without undue reservation.

## Author Contributions

LH, XG, and MF: study concept and design. LH, BN, XG, and LS: acquisition, analysis, or interpretation of data. LH, MF, XG, HW, and HL: drafting of the manuscript. XG: critical revision of the manuscript for important intellectual content. LH and BN: statistical analysis. XG and HW: administrative, technical, or material support. LS: supervision. All authors contributed to the article and approved the submitted version.

## Funding

This work was supported by National Natural Science Foundation of China (grant numbers: 71774005 and 72074007).

## Conflict of Interest

The authors declare that the research was conducted in the absence of any commercial or financial relationships that could be construed as a potential conflict of interest.

## Publisher's Note

All claims expressed in this article are solely those of the authors and do not necessarily represent those of their affiliated organizations, or those of the publisher, the editors and the reviewers. Any product that may be evaluated in this article, or claim that may be made by its manufacturer, is not guaranteed or endorsed by the publisher.

## Abbreviations

AURI: Acute Upper Respiratory Tract Infection
AB: Acute Bronchitis
CAP: Community Acquired Pneumonia
STGs: standard treatment guidelines
ZMDP: Zero Mark-up Drug Policy
CPMP: Centralized Procurement of Medicine Policy
ITS: interrupted time series
GDP: Gross Domestic Product
ATC: Anatomical Therapeutic and Chemical
AMS: Antimicrobial Stewardship.
