# Supplementary material for: The Impact of Sanming Healthcare Reform on Antibiotic Appropriate Use in County Hospitals in China
Source: Front Public Health. 2022 Jun 27;10:936719. doi: 10.3389/fpubh.2022.936719 (PMC9271699; doi:10.3389/fpubh.2022.936719)
Supplement: Supplementary file 1 [file Table_1.DOCX]

**Appendix**

**Appendix 1 Indicators measuring appropriate antibiotic use for Children and Adult with three sample diseases, from 2011 to 2017.**

| **Indicators** | **2011** | **2012** | **2013** | **2014** | **2015** | **2016** | **2017** |
| --- | --- | --- | --- | --- | --- | --- | --- |
| ***Acute Upper respiratory infection*** |  |  |  |  |  |  |  |
| *Children* |  |  |  |  |  |  |  |
| STGs compliance rate (%) | 30.3 | 18.7 | 21.8 | 30.8 | 33.9 | 43.1 | 45.7 |
| Antibiotic prescription rate (%) | 73.3 | 71.1 | 67.9 | 72.5 | 69.4 | 68.9 | 63.5 |
| Combined antibiotic prescription rates (%) | 11.7 | 10.3 | 8.9 | 7.9 | 6.3 | 4.8 | 2.4 |
| *Adult* |  |  |  |  |  |  |  |
| STGs compliance rate (%) | 41.0 | 52.4 | 56.5 | 56.0 | 62.2 | 54.0 | 48.6 |
| Antibiotic prescription rate (%) | 89.1 | 90.7 | 92.1 | 90.2 | 89.5 | 81.4 | 73.5 |
| Combined antibiotic prescription rates (%) | 27.6 | 20.9 | 16.1 | 13.6 | 30.4 | 30.2 | 14.0 |
| ***Acute Bronchitis*** |  |  |  |  |  |  |  |
| *Children* |  |  |  |  |  |  |  |
| STGs compliance rate (%) | 30.9 | 34.1 | 41.1 | 65.6 | 72.7 | 88.9 | 95.1 |
| Antibiotic prescription rate (%) | 86.6 | 87.4 | 89.2 | 85.3 | 83.1 | 84.6 | 84.4 |
| Combined antibiotic prescription rates (%) | 12.0 | 6.7 | 8.7 | 6.5 | 4.0 | 3.1 | 1.1 |
| *Adult* |  |  |  |  |  |  |  |
| STGs compliance rate (%) | 62.2 | 61.9 | 65.2 | 78.9 | 66.9 | 87.4 | 96.2 |
| Antibiotic prescription rate (%) | 96.0 | 95.2 | 94.9 | 92.8 | 90.7 | 89.7 | 92.1 |
| Combined antibiotic prescription rates (%) | 43.4 | 38.1 | 34.8 | 25.9 | 39.6 | 33.0 | 32.4 |
| ***Community Acquired Pneumonia*** |  |  |  |  |  |  |  |
| *Children* |  |  |  |  |  |  |  |
| STGs compliance rate (%) | 33.0 | 24.8 | 12.5 | 33.3 | 40.9 | 53.4 | 69.8 |
| Antibiotic prescription rate (%) | 81.6 | 79.6 | 78.7 | 75.0 | 74.6 | 77.4 | 85.5 |
| Combined antibiotic prescription rates (%) | 10.6 | 20.4 | 29.5 | 16.9 | 18.6 | 12.0 | 16.1 |
| *Adult* |  |  |  |  |  |  |  |
| STGs compliance rate (%) | 27.3 | 26.6 | 24.8 | 28.8 | 36.6 | 71.7 | 84.4 |
| Antibiotic prescription rate (%) | 90.4 | 92.6 | 94.0 | 90.2 | 94.8 | 89.0 | 89.6 |
| Combined antibiotic prescription rates (%) | 60.2 | 64.3 | 68.1 | 64.2 | 62.1 | 56.3 | 43.6 |
